# Evidence of the Prognostic Value of Pretreatment Systemic Inflammation Response Index in Cancer Patients: A Pooled Analysis of 19 Cohort Studies

**DOI:** 10.1155/2020/8854267

**Published:** 2020-08-31

**Authors:** Yi Zhang, Fangteng Liu, Yang Wang

**Affiliations:** ^1^Department of General Surgery, The First People's Hospital of Neijiang, Neijiang, 641000 Sichuan Province, China; ^2^Department of General Surgery, The Second Affiliated Hospital of Nanchang University, Nanchang, 330000 Jiangxi Province, China

## Abstract

**Objective:**

Systemic inflammation response index (SIRI) is a new inflammation-based evaluation system that has been reported for predicting survival in multiple tumors, but the prognostic significance of SIRI in cancers has not been evinced.

**Methods:**

Eligible studies updated on December 31, 2019, were selected according to inclusion criteria, the literature searching was performed in PubMed, Web of Science, Google Scholar, and Cochrane. Hazard ratios (HRs), and 95% confidence intervals (CIs) were extracted and pooled by using Stata/SE 14.1.

**Results:**

11 publications involving 19 cohort studies with a total of 5,605 subjects were included. Meta-analysis results evinced that high SIRI was associated with worse OS (HR = 2.30, 95% CI: 1.87-2.83, *p* ≤ 0.001), poor CSS/DSS (HR = 2.83, 95% CI: 1.98-4.04, *p* ≤ 0.001), and inferior MFS/DFS/PFS/RFS/TTP (HR = 1.88, 95% CI: 1.65-2.15, *p* ≤ 0.001). The association of SIRI with OS was not significantly affected when stratified by diverse confounding factors. It was suggested that tumor patients with high pretreatment SIRI levels would suffer from adverse outcomes.

**Conclusion:**

High SIRI is associated with unfavorable clinical outcomes in human malignancies; pretreatment SIRI level might be a useful and promising predictive indicator of prognosis in cancers.

## 1. Introduction

In recent years, despite the great progress that has been made in diagnosis and treatment technologies and strategies [1], most patients with malignancy remain at risk for recurrence or metastasis and had a short survival. Tumor-related inflammation has been identified as an important hallmark of cancer and plays an important role in tumorigenesis and development [2, 3]; there is also a close interaction between inflammation and cancer biological behaviors [4]. Therefore, to predict the prognosis of cancer patients, using inflammation-related parameters could be a good molecular-based strategy.

Systemic inflammation response index, short for SIRI, is a newly developed inflammation-related biomarker. It is based on three common inflammation-related parameters: peripheral neutrophil, monocyte, and lymphocyte counts, and calculated as follows: neutrophils × monocytes/lymphocyte. This score assessment was first developed by Qi et al. in 2016 [5]. This study reported that SIRI was a noninvasive and simple tool for predicting the survival of patients with advanced pancreatic cancer, and SIRI could be also useful to monitor the status of the local immune response and systemic inflammation in patients [5].

Thereafter, SIRI has been attracted great attention for its predictive value in cancer patients [6–9]. SIRI was reported to be associated with clinicopathological characteristics and prognosis in a variety of tumors [10, 11]. For instance, the higher-SIRI patients with renal cell carcinoma had deeper invasion, advanced N stage, and larger tumor size as well as shorter survival time [10]. And in upper tract urothelial carcinoma, SIRI level was found to be related with pathologic T stage, N stage, and lymphovascular invasion, and the SIRI could also be an independent prognostic indicator for overall survival, cancer-specific survival, and metastasis-free survival among these patients [11].

However, the prognostic value of SIRI on different cancers has not been thoroughly clarified. Therefore, this meta-analysis based on all available data was performed to identify the prognostic role of this novel index as a promising indicator in malignancies.

## 2. Materials and Methods

### 2.1. Literature Retrieval and Study Selection

This work was performed according to the preferred reporting items for systemic reviews and meta-analysis (PRISMA). The literature about the prognostic values of the SIRI in human malignant tumors was retrieved in the following available databases: PubMed, Web of Science, Google Scholar, and Cochrane. The retrieved time was as of December 31, 2019, and only the papers in English were reviewed. The search strategy in PubMed was “((systemic inflammation response index) OR SIRI) AND ((((cancer) OR carcinoma) OR tumor))”. The search strategy in Web of Science was #1 (ALL FIELDS: (cancer) OR ALL FIELDS: (carcinoma) OR ALL FIELDS: (tumor)) AND #2 (ALL FIELDS: (systemic inflammation response index) OR ALL FIELDS: (SIRI)). The search strategy in Google scholar and Cochrane was “systemic inflammation response index”.

### 2.2. Inclusion and Exclusion Criteria

Published papers met all of the following terms were included for combined analysis: (1) focusing on human malignant tumors; (2) studying the relationship between pretreatment SIRI level and clinical outcomes; (3) dividing cases into two groups based on the SIRI cutoff value; (4) reporting the prognostic indexes for SIRI, such as overall survival (OS), cancer-specific survival (CSS), recurrence-free survival (RFS), time to progression (TTP), disease-free survival (DFS), disease-specific survival (DSS), metastatic-free survival (MFS), or progression-free survival (PFS); (5) hazard ratios (HRs) and 95% confidence intervals (CIs) for prognosis were acquirable.

The papers were excluded if they are (1) not original studies, including abstracts, case reports, and review articles, (2) redundant published papers, or (3) not available for survival data of HR.

### 2.3. Data Extraction and Quality Assessment

All items regarding the features of cohort studies were extracted, including the basic data of enrolled studies (the name of first author, publication year, country), the characteristics of patients (cancer type, number of cases, age distribution, gender, primary treatment, stage), the information about SIRI, and prognostic outcomes (cutoff value, determination method, end point, analytic method, follow-up interval).

The HRs and 95% CIs were extracted directly from multivariate cox regression analyses preferentially; otherwise, they were extracted from the Kaplan-Meier survival curves and estimated using Engauge Digitizer version 4.1 (http://markummitchell.github.io/engauge-digitizer/); this method has been validated and applied in many meta-analyses [12–14].

The quality assessment of all selected studies was performed according to the Newcastle-Ottowa Scale [15, 16]. It consists of eight items with three subscales (selection, comparability, exposure); the final score is from 0-9. A study that scored ≥7 was considered high quality.

### 2.4. Statistical Analysis

For the assessment of interstudy heterogeneity, the chi-square test and *I*^2^ were used. Significant heterogeneity was identified with *I*^2^ >50% or *p* < 0.1; then, a random-effects model was employed in this case. Publication bias and sensitivity analysis were also examined when the number of cohorts was ≥10. A *p* value <0.05 was considered a significant statistical difference.

## 3. Results

**Figure 1:**
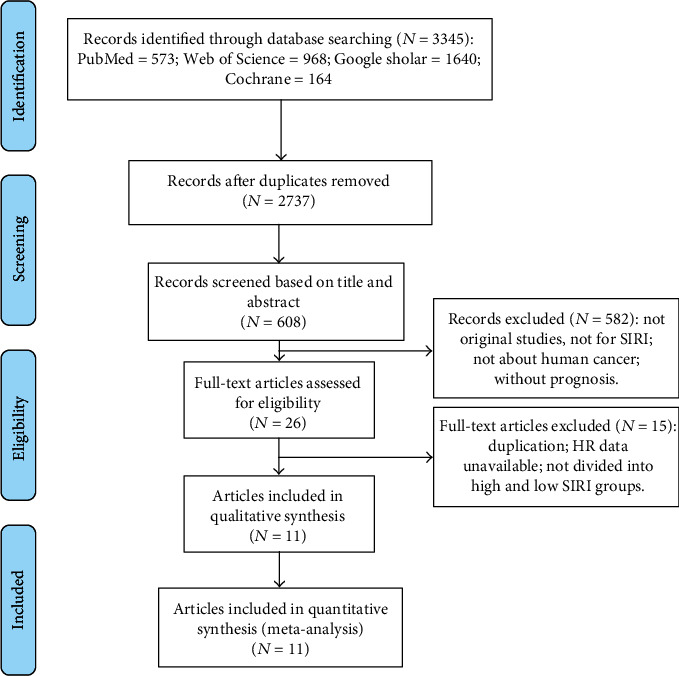
Steps of literature selection in this work.

According to the aforementioned retrieval strategy, a total of 608 potentially relevant publications were initially identified after the removal of duplicate articles. After reviewing the titles and abstracts, 26 articles remained. Then, these articles were carefully checked and selected based on the inclusion and exclusion criteria. Ultimately, a total of 11 papers [5, 10, 11, 17–24] were considered eligible and included for merge-analysis (Figure 1).

These 11 articles were published from 2016 to 2019 with a total of 5,605 subjects, and there were up to 19 cohort studies among these studies, and six articles [10, 11, 17, 18, 20, 24] contained two independent cohorts, and one article [5] included three cohort studies.

Among these studies, twelve cohort studies were based on patients with digestive malignancies, including adenocarcinoma of the oesophagogastric junction (AEG) (1 cohort), pancreatic cancer (PC) (6 cohorts), esophageal squamous cell carcinoma (ESCC) (2 cohorts), gastric cancer (GC) (2 cohorts), and hepatocellular carcinoma (HCC) (1 cohort); and one study was based on nonsmall-cell lung cancer (NSCLC), and two studies were based on nasopharyngeal carcinoma (NPC), upper tract urothelial carcinoma (UTUC), and clear cell renal cell carcinoma (ccRCC), respectively.

All retrospective studies were from China [5, 10, 11, 17–22, 24] except one from Spain [23]. The sample capacity varied from 76 to 542, the demarcation for SIRI ranged from 0.68 to 2.3. All cohort studies enrolled are of high quality (Table 1). For other features of all included cohort studies, such as age distribution, gender, primary treatment, tumor stage, cutoff selection, analytic method of HR, and follow-up, were presented in Table 1.

### 3.1. Impact of SIRI on OS

**Figure 2:**
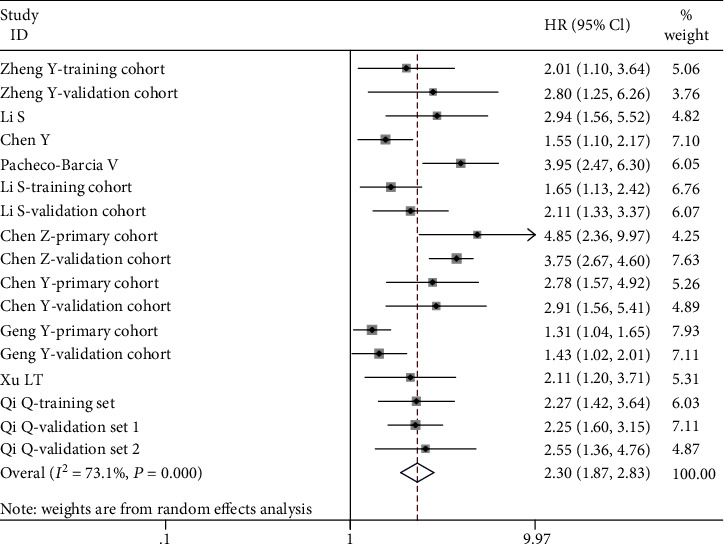
The forest plot for the impact of SIRI on OS in malignant tumors.

A total of 10 published papers consisting of 17 independent cohort studies reported the prognostic role of SIRI for OS, with a total of 4823 cases. On basis of random-effects model (*I*^2^ = 73.1%, *P* = 0.000), the SIRI level was significantly associated with OS (HR = 2.30, 95% CI: 1.87-2.83, *p* ≤ 0.001) (Figure 2). The patients with high SIRI suffered from the shorter OS when compared with subjects with low SIRI.

### 3.2. Impact of SIRI on CSS/DSS

**Figure 3:**
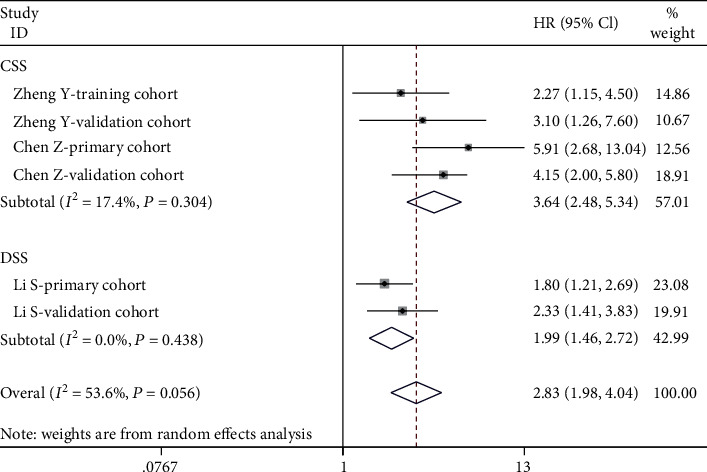
The forest plot for the impact of SIRI on CSS/DSS in malignant tumors.

CSS was available in 4 cohort studies from two papers with 1115 patients; the results showed that SIRI level was negatively correlated with a poor CSS (HR = 3.64, 95% CI: 2.48-5.34, *p* ≤ 0.001); DSS was only available in 2 cohorts from one paper with 782 patients; a significant association was also found between SIRI and DSS (HR = 1.99, 95% CI: 1.46-2.72, *p* ≤ 0.001). Altogether, the group with high SIRI had worse CSS/DSS when compared with the low SIRI group (HR = 2.83, 95% CI: 1.98-4.04, *p* ≤ 0.001) (Figure 3).

### 3.3. Impact of SIRI on MFS/DFS/PFS/RFS/TTP

**Figure 4:**
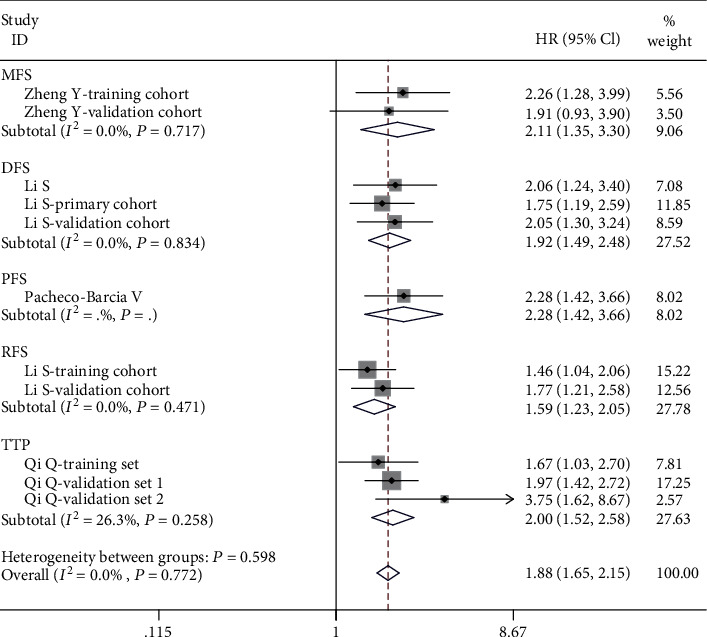
The forest plot for the impact of SIRI on MFS/DFS/PFS/RFS/TTP in malignant tumors.

Similarly, the statistically significant correlation was observed between pretreatment SIRI level and MFS (*n* = two cohort studies with 533 cases, HR = 2.11, 95%CI = 1.35 − 3.30, *p* = 0.001), DFS (*n* = three cohort studies with 1172 cases, HR = 1.92, 95%CI = 1.49 − 2.48, *p* ≤ 0.001), PFS (*n* = one cohort studies with 164 cases, HR = 2.28, 95%CI = 1.42 − 3.66, *p* = 0.001), RFS (*n* = two cohort studies with 681 cases, HR = 1.59, 95%CI = 1.23 − 2.05, *p* ≤ 0.001), and TTP (*n* = three cohort studies with 574 cases, HR = 2.00, 95%CI = 1.55 − 2.58, *p* ≤ 0.001). After combined these data, high SIRI exhibited inferior MFS/DFS/PFS/RFS/TTP (HR = 1.88, 95% CI: 1.65-2.15, *p* ≤ 0.001) based on a fixed effects model (*I*^2^ = 0.0%, *p* = 0.772) (Figure 4).

### 3.4. Stratification Analysis of the Impact of SIRI on OS

For there are 10 publications with up to 17 cohorts (*N* ≥ 10) reporting the relationship between SIRI and OS, and the significant heterogeneity in the combined result, thus, the subgroup analyses were done here, including publishing year, country, sample number, primary treatment, method for cutoff selection, dividing line for SIRI, analytic method for HR, tumor stage, treatment, cancer system, and period of follow-up. As presented in Table 2, despite the different types of variation, a high level of SIRI was significantly associated with shorter OS in human malignant tumors. Nevertheless, no significant heterogeneity (*I*^2^ <50%) could be found the stratification analysis by publishing time (2016-2017), sample capacity <300, X-tile software for cutoff selection, cutoff <1.17, tumor stage (nonmetastatic, mixed), no-surgery therapy, tumor origin from urinary or head and neck, and duration of follow-up <5 years.

### 3.5. Publication Bias

**Figure 5:**
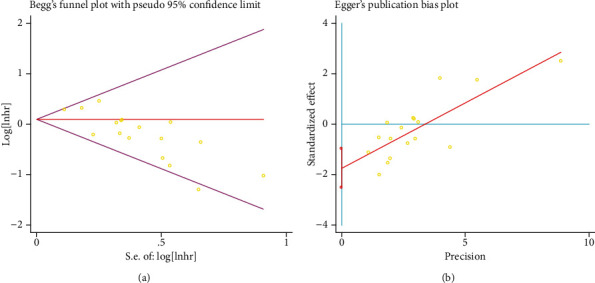
Begg's and Egger's plots for the meta-analysis with OS.

For the meta-analysis with OS, potential publication bias was found by Begg's and Egger's test (*p* ≤ 0.05), as shown in Figure 5. However, the pooled result was demonstrated to be reliable and still significant using the trim and fill method.

### 3.6. Sensitivity Analysis

**Figure 6:**
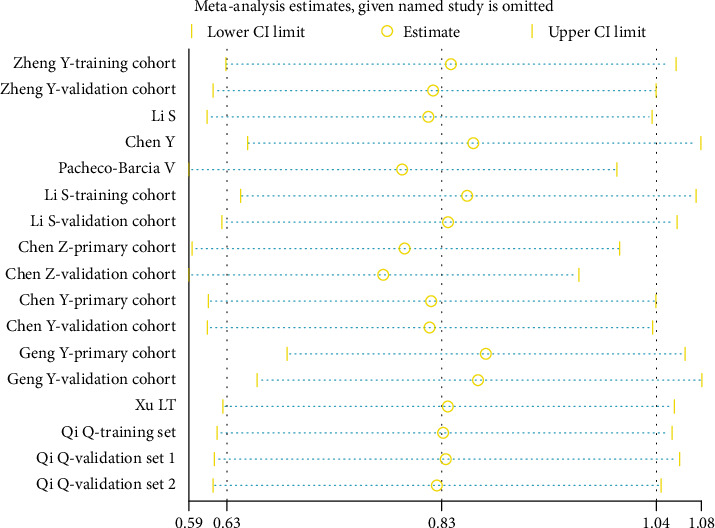
Sensitivity analysis for the correlation of SIRI with OS.

As shown in Figure 6, the pooled results for the correlation of SIRI with OS did not change significantly after the removal of each cohort study; this suggested that the finding was robust.

## 4. Discussion

SIRI is a newly well-established scoring system, which is characterized by a simple and effective integration of three laboratory indicators (neutrophils, lymphocytes, and monocytes); these biomarkers can be easily available and also inexpensive in routine clinical practice. As a promising inflammatory-related index, SIRI could reflect patients' systemic inflammatory response, and the SIRI was also reported to be correlated with liver function parameters, such as aspartate aminotransferase (AST), alanine aminotransferase (ALT), and total bilirubin [19]. And there was a correlation between SIRI and chemotherapy, patients with low SIRI might benefit more from postoperative adjuvant chemotherapy [5].

More notably, SIRI has reported to be superior in prognostic efficiency than other indices, the discriminatory powers of SIRI were significant in survival prediction. In RCC [10], the prognostic value of the SIRI was significantly superior to that of the neutrophil-to-lymphocyte ratio (NLR), platelet-lymphocyte ratio (PLR), monocyte-to-lymphocyte ratio (MLR), and Memorial Sloan Kettering Cancer Center (MSKCC) score in both two independent cohorts. And for NPC and AEG [17, 22], the AUC value was higher for SIRI compared with the NLR, PLR, and MLR at both the 3- and 5-year follow-up. SIRI also achieved the largest AUCs compared with those of NLR, PLR, and lymphocyte-to-monocyte ratio (LMR) and was the only significant discriminator for OS and DFS in the propensity score matching cohort in NSCLC [21]. Besides, SIRI could more accurately predict OS of ESCC patients compared with the TNM staging system in the nomogram [18]. These results proved that the improvement of the ability of survival prediction for SIRI in cancer patients.

In current meta-analysis, 11 publications [5, 10, 11, 17–24] consisting of 19 cohort studies with 5605 malignancy subjects were enrolled. Based on all available data, we firstly evinced that higher SIRI was related to unfavorable prognosis in various malignant tumors. Combined results demonstrated that higher SIRI was associated with poor clinical outcomes, including shorter OS (HR = 2.30, 95% CI: 1.87-2.83, *p* ≤ 0.001), worse CSS/DSS (HR = 2.83, 95% CI: 1.98-4.04, *p* ≤ 0.001), and inferior MFS/DFS/PFS/RFS/TTP (HR = 1.88, 95% CI: 1.65-2.15, *p* ≤ 0.001), this indicated that tumor cases with higher SIRI would suffer from shorten survival rate, increased progression, and recurrence or metastatic rate.

And we also further performed the stratification analysis of the impact of SIRI on OS. The stratified analysis revealed that there was a strong association between higher SIRI and poor OS, though diverse factors, including publication date, country, sample size, cancer system, primary treatment, method for cutoff selection, SIRI dividing line, analytic method for HR, stage, and follow-up varied between different groups. Taking all these findings together, SIRI, as an inflammation-related indicator, could serve as a valuable and powerful biomarker in tumor patients.

Some potential limitations for this pooled analysis must be mentioned and explained. First, the heterogeneity was significant in the analysis with OS. Though no heterogeneity could be found in some subgroup analyses, such as no-surgery therapy and X-tile software for cutoff selection. And another limitation in our work is publication bias, which was found for OS. However, the trim and fill method, as well as sensitivity analysis, showed that the pooled result for OS was robust and reliable. Because of the limited number of articles about other prognostic indexes, such as CSS, DSS, MFS, and DFS, the publication bias and sensitivity analysis were not performed. Lastly, larger clinical studies with high quality from more regions are also required in the future.

## 5. Conclusion

In summary, pretreatment SIRI is related to poor outcomes in human tumors, and pretreatment SIRI could act as a promising predictive indicator of adverse prognosis.

## Figures and Tables

**Table 1:** Main characteristics of the nineteen cohort studies enrolled in this work.

Study information	Year	Country	Cancer type	Total number of cases (M/F)	Age distribution	Primary treatment	Cutoff value	Cutoff selection	Stage	End point	Analytic method	Follow-up interval	NOS
Zheng Y-training cohort [11]	2019	China	UTUC	259 (185/74)	Mean: 67.5 years	With surgery	1.36	ROC analysis	Nonmetastatic	OS, CSS, MFS	Multivariate	Median 33.3 (IQR:15.5-64.2) months	8
Zheng Y-validation cohort [11]	2019	China	UTUC	274 (184/90)	Mean: 65.9 years	With surgery	1.36	ROC analysis	Nonmetastatic	OS, CSS, MFS	Multivariate	Median 44.9 (IQR:26.93-65.8) months	8
Li [21]	2019	China	NSCLC	390 (243/147)	224: ≤65 years; 166: >65 years	With surgery	0.99	ROC analysis	Nonmetastatic	OS, DFS	Multivariate	Median 50 (range 12-66) months	8
Chen [22]	2019	China	AEG	302 (244/58)	Median: 63 years	With surgery	0.68	ROC analysis	Nonmetastatic	OS	Multivariate	Median 55 (range 4-98) months	8
Pacheco-Barcia [23]	2019	Spain	PC	164 (92/72)	Median: 66 years	No surgery	2.3	ROC analysis	Metastatic	OS, PFS	Multivariate	Less than 60 months	7
Li S-training cohort [24]	2019	China	PC	371 (224/147)	Median: 62 years	With surgery	0.69	ROC analysis	Nonmetastatic	OS, RFS	Multivariate	Above 60 months	8
Li S-validation cohort [24]	2019	China	PC	310 (164/146)	Median: 60 years	With surgery	0.69	ROC analysis	Nonmetastatic	OS, RFS	Multivariate	Less than 60 months	7
Chen Z-primary cohort [10]	2019	China	ccRCC	414 (257/157)	Median: 56.3 years	With surgery	1.35	X-tile software	Mixed	OS, CSS	Multivariate	Median 69.2 months, 1 to 151 months	8
Chen Z-validation cohort [10]	2019	China	ccRCC	168 (101/67)	65: >60 years; 103: ≤60 years	With surgery	1.35	X-tile software	Mixed	OS, CSS	K-M	Above 60 months	8
Chen Y-primary cohort [17]	2018	China	NPC	285 (210/75)	106: <50 years; 179: ≥50 years	No surgery	0.84	X-tile software	Mixed	OS	Multivariate	Above 60 months	8
Chen Y-validation cohort [17]	2018	China	NPC	213 (157/56)	86: <50 years; 127: ≥50 years	No surgery	0.84	X-tile software	Mixed	OS	Multivariate	Above 60 months	8
Geng Y-primary cohort [18]	2018	China	ESCC	542 (416/126)	Mean: 54 years	With surgery	1.2	ROC analysis	Nonmetastatic	OS	Multivariate	Above 60 months	8
Geng Y-validation cohort [18]	2018	China	ESCC	374 (280/94)	Mean: 51 years	Withsurgery	1.2	ROC analysis	Nonmetastatic	OS	Multivariate	Above 60 months	8
Li S-primary cohort [20]	2017	China	GC	455 (321/134)	Mean: 57.6 years	With-surgery	0.82	ROC analysis	Non-metastatic	DSS, DFS	Multivariate	Median 77.53 months (range 3.03-111.73)	8
Li S-validation cohort [20]	2017	China	GC	327 (235/92)	Mean: 57.6 years	With surgery	0.82	ROC analysis	Nonmetastatic	DSS, DFS	Multivariate	Median 56.33 months (range 4.9-76.3)	8
Xu LT [19]	2017	China	HCC	183 (155/28)	Mean: 53.7 years	No surgery	1.05	ROC analysis	Mixed	OS	Multivariate	Above 60 months	9
Qi Q-training set [5]	2016	China	PC	177 (108/69)	Mean: 58.8 years	No surgery	1.8	ROC analysis	Mixed	OS, TTP	Multivariate	Less than 60 months	8
Qi Q-validation set 1 [5]	2016	China	PC	321 (208/113)	Mean: 61.0 years	No surgery	1.8	ROC analysis	Mixed	OS, TTP	Multivariate	Less than 60 months	7
Qi Q-validation set 2 [5]	2016	China	PC	76 (46/30)	Mean: 60.9 years	No surgery	1.8	ROC analysis	Mixed	OS, TTP	Multivariate	Less than 60 months	7

UTUC: upper tract urothelial carcinoma; NSCLC: nonsmall-cell lung cancer; AEG: adenocarcinoma of the oesophagogastric junction; PC: pancreatic cancer; ccRCC: clear cell renal cell carcinoma; NPC: nasopharyngeal carcinoma; ESCC: esophageal squamous cell carcinoma; GC: gastric cancer; HCC: hepatocellular carcinoma; OS: overall survival; CSS: cancer-specific survival; RFS: recurrence-free survival; TTP: time to progression; DFS: disease-free survival; DSS: disease-specific survival; MFS: metastatic-free survival; PFS: progression-free survival; M/F: male/female; ROC: the receiver operating characteristic curve; IQR: interquartile range.

**Table 2:** Stratification analysis of the prognostic value of SIRI on OS in malignant tumors.

Subgroup	Number of cohorts	Pooled HR (95% CI)	Heterogeneity		Significance
			*I* ^2^ (%)	*p* value	
Altogether	17	2.30 (1.87-2.83)	73.1	0.000	*p* ≤ 0.001
Publishing year					
2016-2017	4	2.27 (1.80-2.85)	0.0	0.978	*p* ≤ 0.001
2018-2019	13	2.33 (1.79-3.03)	79.6	0.000	*p* ≤ 0.001
Country					
China	16	2.21 (1.80-2.72)	71.2	0.000	*p* ≤ 0.001
Spain	1	3.95 (2.47-6.30)	−	−	*p* ≤ 0.001
Sample number					
<300	9	2.94 (2.47-3.50)	9.6	0.356	*p* ≤ 0.001
≥300	8	1.87 (1.48-2.37)	66.1	0.004	*p* ≤ 0.001
Method for cutoff selection					
ROC analysis	13	2.01 (1.66-2.42)	58.4	0.004	*p* ≤ 0.001
X-tile software	4	3.56 (2.87-4.43)	0.0	0.577	*p* ≤ 0.001
Dividing line for SIRI					
<1.17	7	2.04 (1.67-2.49)	17.8	0.294	*p* ≤ 0.001
≥1.17	10	2.43 (1.77-3.33)	82.5	0.000	*p* ≤ 0.001
Analytic method for HR					
Multivariate	16	2.17 (1.80-2.62)	61.8	0.001	*p* ≤ 0.001
K-M	1	3.75 (2.67-4.60)	−	−	*p* ≤ 0.001
Stage					
Metastatic	1	3.95 (2.47-6.30)	−	−	*p* ≤ 0.001
Nonmetastatic	8	1.68 (1.40-2.01)	34.4	0.153	*p* ≤ 0.001
Mixed	8	2.81 (2.30-3.44)	30.0	0.188	*p* ≤ 0.001
Treatment					
With surgery	10	2.13 (1.58-2.87)	81.2	0.000	*p* ≤ 0.001
No surgery	7	2.58 (2.15-3.11)	0.0	0.571	*p* ≤ 0.001
Cancer system					
Urinary	4	3.28 (2.34-4.60)	35.5	0.199	*p* ≤ 0.001
Respiratory	1	2.94 (1.56-5.52)	−	−	*p* ≤ 0.001
Digestive	10	1.93 (1.57-2.38)	64.3	0.003	*p* ≤ 0.001
Head and neck cancer	2	2.84 (1.86-4.32)	0.0	0.916	*p* ≤ 0.001
Follow-up					
< 5 years	5	2.51 (2.02-3.13)	14.8	0.320	*p* ≤ 0.001
≥5 years	12	2.22 (1.69-2.91)	78.4	0.000	*p* ≤ 0.001

## Data Availability

The data used in the meta-analysis are from previously published studies, which have been cited in the text.
